# Memorial Sloan-Kettering Cancer Center: Two Decades of Experience with Ductal Carcinoma In Situ of the Breast

**DOI:** 10.1155/2012/723916

**Published:** 2012-05-23

**Authors:** Daniel Xavier Choi, Kimberly J. Van Zee

**Affiliations:** Breast Service, Department of Surgery, Memorial Sloan-Kettering Cancer Center, 300 East 66th Street, 8th Floor, New York, NY 10065, USA

## Abstract

Researchers at Memorial Sloan-Kettering Cancer Center have investigated many aspects of their experience with ductal carcinoma in situ of the breast over the past 20 years. This paper summarizes the most clinically relevant findings.

## 1. Introduction

Since its founding in 1884 as the New York Cancer Hospital, Memorial Sloan-Kettering Cancer Center (MSKCC) in New York, NY, has concentrated its clinical and research efforts on the diagnosis and management of cancer. This review summarizes MSKCC's work on ductal carcinoma in situ (DCIS) of the breast. 

## 2. Mammographic Features of DCIS and Its Recurrences

In the late 1980s and early 1990s, increasing screening mammography resulted in increasing detection of clinically occult carcinomas, including DCIS. From 1973 to 1992, the incidence of DCIS increased by 557%, from 2.4 to 15.8 per 100,000 women [1]. Prior to widespread screening mammography, DCIS constituted 0.8% to 5.0% of all breast carcinomas; by 1996 it constituted 14% [2, 3].

In these early days, when the diagnosis of DCIS was exponentially increasing, Dershaw et al. attempted to define the mammographic features of DCIS. They retrospectively reviewed mammograms, specimen radiographs, and pathology reports for 51 patients with DCIS treated in the 1980s [4]. DCIS was radiographically evident as microcalcifications alone in 68% and as microcalcifications within a mass in 30%. Several radiographic findings indicated multifocal disease: mass larger than 2.5 cm; more than one mass; more than one cluster of microcalcifications; or parallel linear intraductal calcifications. This work demonstrated that in the overwhelming majority of patients, DCIS presents as mammographically evident microcalcifications and that specific mammographic findings may suggest multifocal disease.

During the 1980s and 1990s, breast surgery for DCIS became progressively less invasive. No randomized trial was ever performed comparing breast conservation to mastectomy for DCIS. However, as results from Fisher et al. [5] and Veronesi et al. [6], which proved that breast-conserving surgery (BCS) plus radiation was equivalent to total mastectomy (TM) for invasive cancer, were assimilated, it was assumed that the same findings would hold for DCIS. In 1983, 71% of patients with DCIS were treated with TM, and by 1992, only 44% were [1].

TM eliminates the need for postoperative screening mammography; BCS does not—the remaining breast requires continued surveillance for ipsilateral breast tumor recurrences (IBTRs). This was the focus of investigation of Liberman et al. [7]. They performed a retrospective review of 162 patients treated with BCS for DCIS between 1978 and 1990. Median follow-up was 75 months (range, 3–210 months). Thirty-three (20%) were diagnosed with IBTRs; of these, mammograms were available for review in 20. Median time to IBTR was 26 months (range, 6–168 months); IBTRs were DCIS-only in 13 (65%), and in-situ and invasive ductal carcinoma in 7 (35%). They found that: (1) 95% of IBTRs were mammographically evident (85% detectable only mammographically; 10% detectable mammographically and by palpation; 5% detectable only by palpation); (2) IBTRs were mammographically evident whether they were DCIS or invasive; and (3) 90% of IBTRs demonstrated calcifications, of which 79% demonstrated a mammographic pattern identical to the primary tumor (clustered versus linear versus multiple versus regional versus segmental), and of which 82% demonstrated a mammographic morphology identical to the primary tumor (linear versus pleomorphic versus punctate). Liberman et al. definitively demonstrated that mammography can detect IBTRs in patients who undergo BCS for DCIS.

## 3. Sentinel Lymph Node Metastasis in Patients with DCIS: Incidence and Clinical Implications

Cody et al. [8] and Cody and Van Zee [9] ask: “Is axillary node staging required in patients with DCIS of the breast?” Its nomenclature would suggest that DCIS is incapable of breaking through the ductal basement membrane, of accessing breast parenchyma or lymphatic channels, and of spreading locally, regionally, or distantly. In fact, Lagios and Silverstein argue that sentinel lymph node biopsy (SLNB) is “dangerous and unwarranted” in the setting of DCIS [10]. They are correct: searching for metastatic spread of a lesion that is by definition incapable of metastatic spread may yield potential problems—one may find nothing, thereby wasting effort, time, and health care resources, or one may induce harm by causing morbidity, including the most feared complication of axillary surgery: lymphedema [11, 12]. In a sense, Cody and Van Zee agree with Lagios and Silverstein. They argue that “the strongest argument for sentinel lymph node (SLN) biopsy in DCIS is the diagnostic uncertainty and inherent sampling error of conventional pathologic techniques. SLN biopsy is indicated in any DCIS patient who may have an underlying invasive cancer, especially those who require mastectomy” [9]. Two studies from MSKCC attempt to evaluate these arguments.

Klauber-DeMore et al. performed a clinical audit of 76 patients with DCIS and 38 patients with DCIS with microinvasion (DCIS-MI), all of whom underwent SLNB between 1997 and 1999 [13]. “Patients with DCIS were considered to be at high risk and were selected for SLN biopsy if there was sufficient concern that an invasive component would be identified in the specimen during the definitive surgery.” These high-risk patients had a palpable mass (21%), mammographic mass (34%), histology suspicious but not diagnostic for microinvasion (24%), multicentric disease requiring TM (53%), or high nuclear grade or non-high nuclear grade with necrosis (72%). DCIS-MI was in and of itself high-risk.

For patients with DCIS, 9 (12%) had a positive SLN. Seven of these patients had isolated tumor cells positive only by immunohistochemistry—what is currently categorized as pN0(i+) according to the subsequently revised American Joint Commission on Cancer (AJCC) staging system, 6th Edition (2002) and 7th Edition (2010). Upon completion axillary lymph node dissection, 1 patient had additional macrometastasis (>2 mm) positive by routine hematoxylin and eosin (H&E). These findings prompted re-evaluation of primary-tumor specimens. One patient had a focus of microinvasion, one patient had a metastatic invasive carcinoma of the contralateral breast, and 2 patients had lymphovascular invasion despite no evidence of stromal invasion.

For patients with DCIS-MI, 3 (10%) had a positive SLN: one macrometastasis positive by routine H&E; one micrometastasis positive by routine H&E and immunohistochemistry; and one with isolated tumor cells positive by immunohistochemistry. Upon completion axillary lymph node dissection, no further positive nodes were found. Re-evaluation of primary-tumor specimens revealed invasion greater than 1 mm in 2 patients, changing the tumor status from T*is* to T1mi. Furthermore, one of these patients demonstrated in-breast lymphatic invasion.

Klauber-DeMore et al. demonstrated that the frequencies of positive axillary SLNs, including isolated tumor cells in patients with DCIS in whom the surgeon was concerned that invasion was present and in patients with DCIS-MI, were 12% and 10%, respectively [13]. Moore et al. sought to evaluate the clinical relevance of such involvement [14]. Between 1994 and 2002, 2159 patients underwent surgery for DCIS at MSKCC, the John Wayne Cancer Institute, or the University of Southern California. Of these, 470 patients underwent SLNB. As in the previous report, for the most part, these patients had high-risk DCIS.

Forty-three (9%) had a positive SLN. Of these, 3 (7%) had macrometastases, 4 (9%) had micrometastases, and 36 (84%) had isolated tumor cells. Re-evaluation of primary-tumor specimens demonstrated that 2 (5%) had DCIS-MI and 2 (5%) had lymphovascular invasion. Ultimately, 9 of 43 (21%) high-risk DCIS patients with a positive SLN, and 9 of 470 (2%) of all high-risk DCIS patients were upstaged to stage 1 or stage 2 (AJCC 6th Edition) as a direct result of SLNB. Based on these findings, 16 patients (37%) received chemotherapy. At median follow-up of 27 months (range, 3–88 months), one patient with isolated tumor cells in her SLN developed hepatic metastases and died of disease. Re-evaluation of her primary-tumor specimen demonstrated previously unidentified microinvasion. Finally, Moore et al. found 2 factors associated with SLN positivity for patients with DCIS: extensive disease requiring mastectomy (12% versus 4%, *P* = 0.016) and presence of necrosis (11% versus 3%, *P* = 0.04).

In summary, these 2 reports conclude that approximately 1 of 10 patients with high-risk DCIS or DCIS-MI have evidence of axillary metastases on SLNB, including macro- or micrometastases, and isolated tumor cells. And of these, only about 20% are considered upstaged according to the AJCC 6th Edition staging system (and 26% according to the AJCC 7th Edition staging system). Therefore, only about 2% of all women with high-risk DCIS or DCIS-MI are upstaged due to SLN findings. In conclusion, while SLNB should not be routinely used for DCIS, it is appropriate in women undergoing TM or in women in whom the suspicion of invasive carcinoma is high.

## 4. Utility of Preoperative MRI for Paget's Disease of the Breast

Paget's disease of the breast usually presents with eczematous changes of the nipple-areola complex, such as erythema, scaling, and itching [15]. In-situ or invasive carcinoma usually underlies these epidermal changes [16]. Thus, upon clinical or pathologic diagnosis, breast imaging is pursued in an effort to characterize the extent of the underlying associated cancer. Mammography has been the mainstay of radiologic work-up. Morrogh et al. sought to evaluate the utility of MRI as a complementary imaging modality [17].

The authors retrospectively reviewed the charts of 34 patients who presented between 1995 and 2005 with changes in the nipple-areola complex consistent with Paget's disease [17]. Patients with a prior history of ipsilateral breast carcinoma were excluded. All 34 patients underwent preoperative mammography; 13 underwent preoperative MRI. All underwent surgery, and, on final pathology, 32 of 34 patients (94%) had an underlying cancer (DCIS, 56%; DCIS-MI, 18%; invasion, 21%) and 2 of 34 patients (6%) had an underlying benign lesion (atypical ductal hyperplasia, 3%; or intraductal papilloma, 3%).

Preoperative mammography identified 11 of 32 (34%) cancers, accurately demonstrating extent of disease in 9. Among the 13 women who underwent MRI, MRI identified 7 of 12 (58%) cancers, accurately demonstrating extent of disease in 6. After a positive mammogram, subsequent MRI did not alter course of treatment. However, after a negative mammogram, subsequent MRI detected otherwise-occult disease in 4 of 8 patients, accurately demonstrating extent of disease in all. Overall, mammography had 100% positive predictive value, 9% negative predictive value, 34% sensitivity, and 100% specificity, while MRI had 100% positive predictive value, 17% negative predictive value, 58% sensitivity, and 100% specificity.

In this series, there was no benefit of MRI in patients with positive mammography. However, in patients with negative mammography, MRI demonstrated an underlying occult cancer and the extent of that cancer in about half, thus providing a diagnosis and facilitating surgical treatment. The ability to determine extent of disease was tantamount, for it enabled the surgeon to determine the feasibility of BCS. In this study, 59% of patients had disease confined to the central part of the breast. Thus for patients with Paget's disease of the breast and a negative mammogram, MRI is helpful in detecting the presence and extent of occult, underlying carcinomas.

## 5. Age: Associations with Local Recurrence of DCIS and with DCIS Treatment Practice Patterns

Early after the publication of randomized studies proving the equivalency of BCS with adjuvant radiotherapy and TM for invasive carcinoma, clinicians and patients slowly adopted BCS for DCIS. Van Zee et al. investigated MSKCC's early experience with BCS for DCIS [18]. They performed a retrospective analysis of 157 patients with DCIS treated with BCS between 1978 and 1990 with a median follow-up of 74 months. Of 157 patients, 33 (21%) were diagnosed with IBTRs. The actuarial IBTR rate was 16.1% at 6 years. Among those who received radiotherapy, the 6-year IBTR rate was 9.6%; without radiotherapy, it was 20.7% (*P* = 0.05). On univariate analysis, clinicopathologic features associated with lower IBTR rates were older age, noncomedo subtype, lower nuclear grade, negative margins, and adjuvant radiotherapy. For patients less than 40 years old, the 6-year IBTR rate was 47.2%; for patients 40–69 years old, the 6-year IBTR rate was 14.0%; for patients 70 years old or greater, the 6-year IBTR rate was 10.8%. Compared to younger patients, lower IBTR rates were seen in older patients whether adjuvant radiotherapy was used or not. IBTR rates with and without radiotherapy for the 3 age groups were <40 years old: 33.3% and 59.2%; 40–69 years old: 8.5% and 19.1%; ≥70 years old: 0.0% and 14.4%, respectively. Compared to younger patients, older patients tended to have lower nuclear grades and were less likely to receive adjuvant radiotherapy, but no factor (including clinical presentation, tumor size, comedo subtype, nuclear grade, presence of necrosis, margin status, or adjuvant radiotherapy) demonstrated a statistically significant association with age. This study showed that compared to older patients, younger patients treated with BCS for DCIS are more likely to develop IBTRs and that they are at a relatively high risk for IBTRs.

This finding, which had not been previously shown for DCIS, has also been observed by Veronesi et al. for invasive carcinoma. He found that women who underwent quadrantectomy alone for invasive carcinomas had an IBTR rate of 17.5% if they were 45 years of age or younger, and 3.8% if they were 55 years of age or older [19]. Veronesi et al. surmised that with age, “the complex structure of the mammary gland disappears and the breast is reduced to a fatty organ with scattered islands of fibroepithelial tissue, without connections between them.”

BCS attempts to balance oncologic and cosmetic imperatives—to treat cancer while maintaining the size and shape of the native breast. Ideally, a surgeon excises a primary tumor to clear margins and leaves as much normal tissue intact as possible. In a sense, TM allows for the biggest possible margin and, in doing so, achieves oncologic imperatives, but pays the cosmetic price of complete removal of the breast. Conversely, a limited tumorectomy allows for the smallest possible margin and, in doing so, achieves cosmetic imperatives, but is associated with higher rates of IBTR. This may have been particularly true prior to the 1980s, when diligent microscopic assessment of inked margins was not standard.

Hwang et al. postulated that volume of surgical resection may be related to age, which at least partially explains the observation that older women have lower IBTR rates compared to younger women [18, 20]. Older women tend to have higher BMIs and larger breasts, perhaps allowing surgeons to do wider resections. Furthermore, it may be possible that the oncologic-cosmetic balance of BCS is tipped toward wider margins and away from cosmesis in older patients compared to younger patients.

Hwang et al. hypothesized that surgeons were more willing to take larger volumes of tissue in older patients compared to younger patients, increasing IBTR-free survival but possibly compromising cosmesis. They retrospectively reviewed the same population studied by Van Zee et al. and found 126 cases with available information regarding the volume of resection. They found that while tumor size, margin status, histologic subtype, nuclear grade, and size did not correlate with volume of resection, patient age did—younger patients had significantly smaller volumes of resection than older patients (*P* = 0.03). Patients with smaller volumes of resection had higher 6-year IBTR rates than patients with larger volumes of resection (21% versus 5.6%; *P* = 0.16). Also, patients with smaller volumes of resection were more likely to undergo adjuvant radiotherapy than patients with larger volumes of resection, though both groups benefited. For the small volume-of-resection group, the IBTR rate was 12.7% with adjuvant radiotherapy and 29.1% without adjuvant radiotherapy. For the large volume-of-resection group, the IBTR rate was 0% with adjuvant radiotherapy and 7.1% without adjuvant radiotherapy.

In summary, Hwang et al. conclude that older patients tend to have larger volumes of resection, which is associated with lower rates of IBTR, even in the absence of adjuvant radiotherapy. There are 3 possible reasons for this. First, “smaller resection volumes may be used in younger patients to achieve a better cosmetic result, possibly contributing to a high local recurrence” [20], showing that the oncologic-cosmetic balance is tipped away from oncologic imperatives toward cosmetic ones. This had been suggested by the Joint Center for Radiation Therapy for women with invasive breast cancer [21]. Second, as stated previously, in older patients, “the complex structure of the mammary gland disappears and the breast is reduced to a fatty organ with scattered islands of fibroepithelial tissue, without connections between them” [19]; the older breast is anatomically and physiologically less likely to have a diffuse distribution of DCIS and therefore less prone to have unresected microscopic disease. Third, the biology of breast cancer in younger patients is intrinsically more aggressive than the biology of breast cancer in older patients; compared to younger patients, older patients tend to have low nuclear grades and hormone-receptor positivity [20, 22, 23].

Ho et al. expand upon the aforementioned works by Van Zee et al. and Hwang et al. [18, 20] and “aimed to determine the impact of increasing age and other clinicopathologic features on treatment patterns and outcomes in older women with DCIS” [24]. The analysis of Van Zee et al. included 157 patients (of any age) treated with BCS for DCIS over a 12-year period. The analysis of Ho et al. included 646 patients (60 years of age or older) treated between 2000 and 2007 (2 decades after those studied by Van Zee et al. and Hwang et al.), of whom over 75% underwent BCS. Though reasons for this increase are certainly multiple, it is clear that over the past 20 years, widespread screening mammography has resulted in more frequent detection of DCIS and that more and more patients are being treated with BCS than with TM. Ho et al. included 646 patients, despite restricting their study population to those 60 years old or older. This supports the notion that the United States population at large, and cancer patients in particular, are aging. In 2010, 25,000 women 65 years of age or older were diagnosed with DCIS; it is estimated that, in 2030, 39,000 women 65 years old or older will be diagnosed with DCIS, a 56% increase [25].

Ho et al. found that even among older patients, the oldest received less aggressive therapy—for patients aged 60–69 years, 45% received TM, 25% received BCS with radiotherapy, and 30% received BCS alone; for patients aged 70–79 years, 38% received TM, 20% received BCS with radiotherapy, and 40% received BCS alone; for patients aged 80 years or more, 16% received TM, 13% received BCS with radiotherapy, and 71% received BCS alone (*P* < 0.001). In addition to younger age (*P* < 0.001), higher grade (*P* < 0.001) and the presence of necrosis (*P* < 0.01) were associated with TM or radiotherapy.

Median follow-up was 54 months (range, 6–112 months). The 4-year local recurrence rate was 3.6% and differed according to treatment. None of the TM group, 4% of the BCS-radiotherapy group, and 5% of the BCS-alone group experienced a local recurrence at 4 years (*P* < 0.005). When the BCS-radiotherapy and BCS-alone groups were compared, no statistically significant differences in local recurrence were detected (*P* = 0.66). Besides treatment, no other factors—including age—were associated with local recurrence.

The study of Ho et al. is neither randomized nor controlled. Rather, it is a clinical audit; its data are the end-results of innumerable individualized medical decisions. Most patients with BCS did not receive radiotherapy in spite of data from 4 randomized controlled trials that definitively show a lower IBTR rate among those that receive adjuvant radiotherapy [26–29]. Nonetheless, the omission of adjuvant radiotherapy did not translate into substantially different IBTR rates, at least in this patient population. That older women were less likely to experience an IBTR—despite not undergoing adjuvant radiotherapy—suggests that clinicians are able to individualize treatment appropriately. These findings also demonstrate that, in the current era, older women have lower rates of IBTR, confirming the findings of Van Zee et al. and Hwang et al. [18, 20].

## 6. Association of Lobular Neoplasia with Local Recurrence of DCIS

Rudloff et al. sought to determine whether the associated histologic findings of columnar cell changes, atypical ductal hyperplasia, or lobular neoplasia affected IBTR rates in patients who underwent BCS for DCIS [30]. They state that “the presence of concurrent proliferative lesions associated with in situ or infiltrating breast carcinoma may reflect underlying gene perturbations of cancer-related pathways in the uninvolved ductal epithelium, which could be markers of disease risk, occult disease, or, also, the tissue response to an existing tumor.” This study included 294 patients with DCIS treated with wide local excision, with or without radiotherapy, between 1991 and 1995, and included re-review of all available pathology slides. Median follow-up was 11 years (range, 0–16 years). Columnar cell changes were present in 71 (24%), atypical ductal hyperplasia in 37 (13%), and lobular neoplasia in 41 (14%). Fifteen of 41 patients (37%) with lobular neoplasia developed an IBTR; 40 of 227 patients (18%) without lobular neoplasia developed an IBTR (hazard ratio, 2.49; *P* = 0.001). On multivariate analysis, 5-year, 10-year, and 15-year IBTR rates were twice as high in women with DCIS and associated lobular neoplasia (26%, 36%, and 55%, respectively) compared to women with DCIS alone (14%, 19%, and 24%, respectively) (*P* = 0.002). In fact, the presence of lobular neoplasia and the omission of adjuvant radiotherapy demonstrated similar increases in IBTR rates. Columnar cell changes and atypical ductal hyperplasia did not demonstrate increased IBTR rates (*P* = 0.44 and *P* = 0.20, respectively). This paper found that the presence of lobular neoplasia is associated with increased IBTR rates in patients undergoing BCS for DCIS.

The authors hypothesized that "concurrent lobular neoplasia may be a phenotypic manifestation of more extensive genetic abnormalities, resulting in a higher risk for local recurrence." They called for assessment of the surrounding epithelium to further evaluate these findings in future clinical studies of BCS for DCIS. Furthermore, the authors suggested that if patients with DCIS and concurrent lobular neoplasia are at higher risk, they may particularly benefit from the use of risk-reducing adjuvant therapies.

## 7. Association of Margin Width and Volume of Disease Near Margin with Local Recurrence of DCIS

Silverstein et al. found that the IBTR rate was 3% at 8 years for women who had margins at least 1 cm in width and therefore proposed that radiotherapy could be omitted in women with DCIS who undergo BCS and have very wide margins [31, 32]. To test this hypothesis, Rudloff et al. further examined their previous exhaustively characterized population of 294 patients [33]. They had a median follow-up of 11 years. They categorized patients into the same groups used by Silverstein et al.: close or positive (margin width <1 mm), intermediate (margin width =1–9 mm), and widely clear (margin width ≥10 mm). Furthermore, Rudloff et al. quantified the number of ducts involved with DCIS at this margin width as 1 or ≥2. They combined these 2 characteristics into a measure of volume of disease near the margin, such that each patient was categorized as: (1) widely clear margin ≥10 mm, 0 involved ducts within 10 mm of margin; (2) margin width 1 to 9 mm, 1 involved duct at the closest margin; (3) margin width <1 mm, 1 involved duct at the closest margin; and (4) margin width <10 mm, ≥2 involved ducts at the closest margin.

Rudloff et al. found that women with DCIS presumed to be at higher risk of IBTR, including those with higher nuclear grade or necrosis, were more likely to receive radiation than those presumed to be at lower risk (*P* < 0.009). Nonetheless, women receiving radiotherapy had lower rates of IBTR (10-year rates without radiotherapy, 28% versus with radiotherapy, 12%; *P* = 0.002), which is consistent with randomized controlled trials [26, 34]. While adjuvant radiotherapy benefited all populations, it benefited those with closer margins more than those with wider margins. Adjuvant radiotherapy “reduced adjusted IBTR rates by 62% (*P* = 0.002) for all patients; by 83% for lesions with <1 mm margins (*P* = 0.002), by 70% for 1 to 9 mm (*P* = 0.05), and by 24% (*P* = 0.55) for ≥10 mm.” In other words, among patients who did not receive radiotherapy, margin status was significantly associated with IBTR (*P* = 0.02), but receipt of radiotherapy negated any influence of margin width on the risk of IBTR (*P* = 0.66). Because in this series, radiotherapy was given according to clinical judgment in a non-randomized fashion, Rudloff et al. went on to perform a multivariate analysis to adjust for the many other factors that could influence the association between margin width and added value of radiotherapy in reducing IBTR rates (age, palpable mass, lobular neoplasia, nuclear grade, necrosis, endocrine therapy, and number of involved ducts at closest margin). It was unchanged.

Rudloff et al. then proceeded to analyze the association of volume of disease near margin, incorporating margin width and number of involved ducts at the closest margin with IBTR. Among those not receiving radiotherapy, they found that the higher the volume of disease near the margin, the greater the risk of IBTR, with a hazard ratio of 3.37 (*P* = 0.002) for IBTR among patients who had ≥2 involved ducts and <10 mm margin width. However, among those receiving radiotherapy, there was no increased risk of IBTR. Stratified by volume of disease near margin, they showed that the risk reduction associated with radiotherapy was the greatest in those with the highest volume of disease near the margin (hazard ratio of radiotherapy to no-radiotherapy, 0.14; *P* = 0.004) as compared to those with widely clear margins (0 involved ducts within 10 mm of margin; hazard ratio of radiotherapy to no-radiotherapy, 0.60; *P* = 0.39).

Like others [35, 36], Rudloff et al. were unable to identify a subgroup with a very low rate of IBTR without radiotherapy; the 10-year IBTR rate for their most favorable subgroup (patients with wide margins and older age, and without a palpable mass or lobular neoplasia) was 13%. Interestingly, this rate is comparable to the IBTR rate observed in the population that did receive radiotherapy in the Rudloff study (12%) and among the populations that received radiotherapy in randomized controlled trials (9–20% at 8–17 years) [28, 34, 37, 38]. These findings support the concept that the added benefit of radiotherapy varies among DCIS patients of different risk; in other words, the benefit-to-risk ratio is not the same for all DCIS patients. For women with a higher volume of disease near the margin, radiotherapy is associated with a greater risk reduction of IBTR.

## 8. Integrating Clinical and Treatment Factors to Estimate Risk of Local Recurrence of DCIS

For patients undergoing BCS for DCIS, randomized controlled trials demonstrate that adjuvant radiotherapy and hormonal therapy reduce IBTR rates by 50% and 30%, respectively [26, 34, 38]. However, because these treatments are not without risk and because there is no observed survival benefit associated with either, expert clinicians do not support their ubiquitous utilization [39, 40]. Some populations may be at sufficiently low risk of IBTR such that the benefit-to-risk ratio of adjuvant treatment is not justified. Rudloff et al. argue that “the one-size-fits-all approach of adjuvant treatment for all patients with DCIS seems counterintuitive to both the molecular heterogeneity of DCIS as well as the increasing trend toward individualized cancer treatment” [41]. The work of Rudloff et al. discussed above certainly supports the idea that the benefit-to-risk ratio of adjuvant treatment varies with the risk of IBTR for a woman with DCIS. In their next investigation, Rudloff et al. aimed to provide clinicians with the means of providing that “individualized cancer treatment.” Several clinicopathologic factors have been shown to affect IBTRs in patients undergoing BCS for DCIS. These include age, clinical presentation, family history, tumor size, tumor multifocality, margin status, volume of disease at the closest margin, histologic architecture, nuclear grade, and necrosis. Rudloff et al. combine these, as well as treatment factors, to create a clinically useful tool, a nomogram, that integrates the information available in all of these factors in order to estimate risk of IBTR for women undergoing BCS for DCIS. To build this nomogram, Rudloff et al. evaluated 1681 patients treated with BCS for DCIS between 1991 and 2006. Median follow-up was 5.6 years (range, 0–17.5 years). Actuarial 5-year and 10-year IBTR rates were 9% (95% confidence interval (CI) 8–11%) and 15% (95% CI, 13–18%), respectively.

The nomogram includes 10 parameters (age at diagnosis, family history, initial presentation, radiotherapy, endocrine therapy, nuclear grade, necrosis, margins, number of excisions, and year of surgery), which are assigned a score between 0 and 100. The sum of these 10 individual scores is used to determine the 5- and 10-year risk of IBTR. Rudloff et al. provide examples: “The nomogram predicts that a 70-year-old woman (30 points) without family history of breast cancer (0 points), with screen-detected (0 points) DCIS with low nuclear grade (0 points), no necrosis (0 points), and negative margins (0 points) after one re-excision (0 points) who does not undergo RT (100 points) or anti-estrogen therapy (76 points) has a lower than 10% risk (total points: 206) of developing a recurrence within 10 years of BCS. Conversely, a 40-year-old woman (75 points) with a family history of breast cancer (30 points) and DCIS detected as a palpable mass (34 points), who underwent one excision (0 points) resulting in close margins (56 points), with high nuclear grade (27 points) and necrosis (13 points), and who defers RT (100 points) and endocrine therapy (76 points), has about a 50% risk (total points: 411) of developing an IBTR by 10 years.”

The nomogram was internally validated with bootstrapping, and was recently validated in independent populations by Collins et al. [42] and Yi et al. [43]. The nomogram utilizes the many discrete factors that have been shown to affect risk of IBTR and combines them into a user-friendly calculator to estimate risk. Using these readily available factors, this tool can assist clinicians in weighing the pros and cons of various surgical options and adjuvant therapies for DCIS.

## 9. Summary

This paper summarizes the investigations of DCIS by the Breast Surgical Service at MSKCC over the past 2 decades. It includes papers investigating the mammographic features of DCIS and its recurrences, the incidence and clinical implications of SLN metastases in patients with high-risk DCIS, the utility of preoperative MRI for Paget's disease of the breast, the associations of age with local recurrence and practice patterns for treatment of DCIS, the association of concurrent lobular neoplasia with local recurrence of DCIS, the association of margin width and volume of disease near margin with local recurrence of DCIS, and the creation of a nomogram that incorporates many factors simultaneously to estimate risk of IBTR of DCIS.

## Abbreviations

AJCC: American Joint Commission on Cancer
BCS: Breast-conserving surgery
CI: Confidence interval
DCIS: Ductal carcinoma in situ
DCIS-MI: Ductal carcinoma in situ with microinvasion
H&E: Hematoxylin and eosin
IBTR: Ipsilateral breast tumor recurrence
MSKCC: Memorial Sloan-Kettering Cancer Center
SLN: Sentinel lymph node
SLNB: Sentinel lymph node biopsy
TM: Total mastectomy.
